# Sarcomatoid renal cell carcinoma: MRI features and their association with survival

**DOI:** 10.1186/s40644-023-00535-0

**Published:** 2023-02-15

**Authors:** Monica Cheng, Cihan Duzgol, Tae-Hyung Kim, Soleen Ghafoor, Anton S. Becker, Pamela I. Causa Andrieu, Natalie Gangai, Hui Jiang, Abraham A. Hakimi, Hebert A. Vargas, Sungmin Woo

**Affiliations:** 1 Department of Radiology, Memorial Sloan Kettering Cancer Center, 1275 York Ave, New York, NY 10065 USA; 2 Department of Radiology, Massachusetts General Hospital, Harvard Medical School, Boston, MA USA; 3 Department of Radiology, Lowell General Hospital, 295 Varnum Avenue, Lowell, MA 01854, USA; 4 Institute of Diagnostic and Interventional Radiology, University Hospital Zurich, Zurich, Switzerland; 5 Urology Service, Department of Surgery, Memorial Sloan Kettering Cancer Center, New York, NY USA

**Keywords:** Sarcomatoid, Renal cell carcinoma, Magnetic resonance imaging, Survival, Prognosis

## Abstract

**Objective:**

To evaluate MRI features of sarcomatoid renal cell carcinoma (RCC) and their association with survival.

**Methods:**

This retrospective single-center study included 59 patients with sarcomatoid RCC who underwent MRI before nephrectomy during July 2003–December 2019. Three radiologists reviewed MRI findings of tumor size, non-enhancing areas, lymphadenopathy, and volume (and percentage) of T2 low signal intensity areas (T2LIA). Clinicopathological factors of age, gender, ethnicity, baseline metastatic status, pathological details (subtype and extent of sarcomatoid differentiation), treatment type, and follow-up were extracted. Survival was estimated using Kaplan-Meier method and Cox proportional-hazards regression model was used to identify factors associated with survival.

**Results:**

Forty-one males and eighteen females (median age 62 years; interquartile range 51–68) were included. T2LIAs were present in 43 (72.9%) patients. At univariate analysis, clinicopathological factors associated with shorter survival were: greater tumor size (> 10 cm; HR [hazard ratio] = 2.44, 95% CI 1.15–5.21; *p* = 0.02), metastatic lymph nodes (present; HR = 2.10, 95% CI 1.01–4.37; *p* = 0.04), extent of sarcomatoid differentiation (non-focal; HR = 3.30, 95% CI 1.55–7.01; *p* < 0.01), subtypes other than clear cell, papillary, or chromophobe (HR = 3.25, 95% CI 1.28–8.20; *p* = 0.01), and metastasis at baseline (HR = 5.04, 95% CI 2.40–10.59; *p* < 0.01). MRI features associated with shorter survival were: lymphadenopathy (HR = 2.24, 95% CI 1.16–4.71; *p* = 0.01) and volume of T2LIA (> 3.2 mL, HR = 4.22, 95% CI 1.92–9.29); *p* < 0.01). At multivariate analysis, metastatic disease (HR = 6.89, 95% CI 2.79–16.97; *p* < 0.01), other subtypes (HR = 9.50, 95% CI 2.81–32.13; *p* < 0.01), and greater volume of T2LIA (HR = 2.51, 95% CI 1.04–6.05; *p* = 0.04) remained independently associated with worse survival.

**Conclusion:**

T2LIAs were present in approximately two thirds of sarcomatoid RCCs. Volume of T2LIA along with clinicopathological factors were associated with survival.

**Supplementary Information:**

The online version contains supplementary material available at 10.1186/s40644-023-00535-0.

## Introduction

Renal cell carcinoma (RCC) is the eighth most common type of cancer in the United States, with a projection of approximately 79,000 new cases in 2022 [1]. Sarcomatoid RCC is a rare (4–5%) form of RCC that contains pleomorphic spindle cells [2–5]. Sarcomatoid differentiation may develop within any subtype of RCC and is highly aggressive, often presenting as advanced or metastatic disease with median survival of less than 1 year [5, 6]. Despite recognition of sarcomatoid differentiation being associated with poorer outcomes, diagnostic and therapeutic advancements have been limited partly due to its rarity [7].

Magnetic resonance imaging (MRI) has recently gained interest in characterizing renal masses [8, 9]. A few recent studies attempted to identify MRI features that can predict sarcomatoid differentiation. Takeuchi et al. [10, 11] suggested that sarcomatoid RCC may be distinguished from non-sarcomatoid RCC on T2-weighted imaging (T2WI) by identifying an area of lower intensity compared to the contralateral renal cortex (i.e., T2 low signal intensity area [T2LIA]) which corresponded to the sarcomatoid component on pathology. Although subsequent studies have demonstrated some value of this, it has not been established for preoperative diagnosis related to many limitations including small sample size and underestimation of the extent of sarcomatoid differentiation on MRI compared with pathology [12–14]. Other MRI findings have also been reported, however are nonspecific and can be seen in any high-grade tumor (e.g., large size, necrosis, and locally-advanced or metastatic disease) [11, 12].

Recently, a few studies reported that the volume of sarcomatoid component on pathology was associated with survival in patients with RCC [15–17]. In one study, a 10% increase in sarcomatoid component was associated with a 6% increased risk of cancer-related death [16]. However, determining the exact proportion of sarcomatoid differentiation may be difficult even on pathology in these large and often necrotic/hemorrhagic tumors. We hypothesized that volumetric measurements of T2LIA on MRI may be of prognostic value when considering the recent literature. Therefore, the purpose of this study was to evaluate the MRI features of sarcomatoid RCC including the volume of T2LIA, and their association with survival.

## Methods

### Patients

**Fig. 1:**
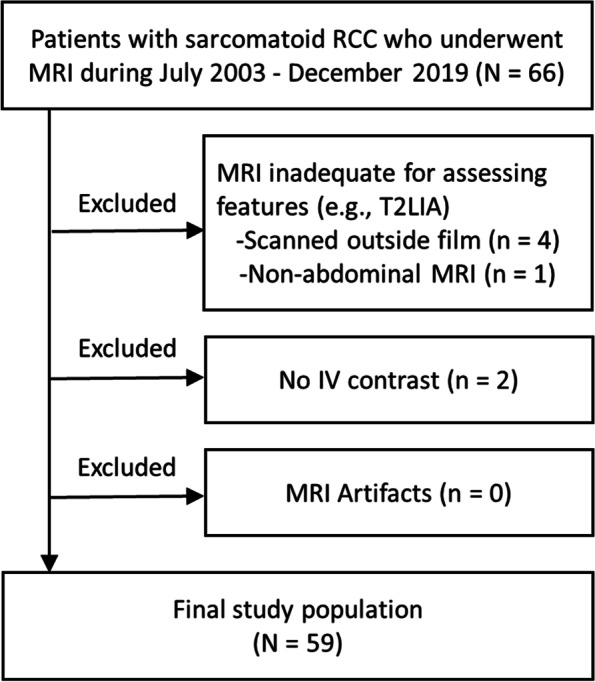
Flowchart of patient inclusion. IV = intravenous; MRI = magnetic resonance imaging; RCC = renal cell carcinoma; T2LIA = T2 low signal intensity area

This retrospective study was performed after obtaining approval from the institutional review board and was compliant with the Health Insurance Portability and Accountability Act. Our pathology database was searched to identify patients with sarcomatoid RCC who underwent MRI during 2003–2019. Exclusion criteria were: (1) MRI inadequate for imaging analysis; (2) lack of intravenous contrast media required to ascertain solid components; and (3) artifacts. Figure 1 illustrates the patient selection process.

### MRI analysis

MRI examinations were performed on 1.5- and 3.0-Tesla scanners. Due to the long inclusion period, the technical parameters varied; however, the axial T2WI used for quantification of T2LIAs were generally obtained with the following: repetition time/echo time, 1500–2400/100–180; slice thickness/interval, 4 cm/4 cm; matrix size, 256 × 192 − 256 × 256; and field-of-view, 30 cm × 30 cm–44 cm × 44 cm. MRIs were reviewed independently by two radiologists specialized in genitourinary oncologic imaging (S.W. and C.D., with 7 and 10 years of post-residency experience, respectively), who were aware that these patients had sarcomatoid RCC but were blinded to other clinical/pathological information; disagreements were adjudicated by a third (H.A.V. with 14 years of subspecialized oncologic imaging experience).

**Fig. 2:**
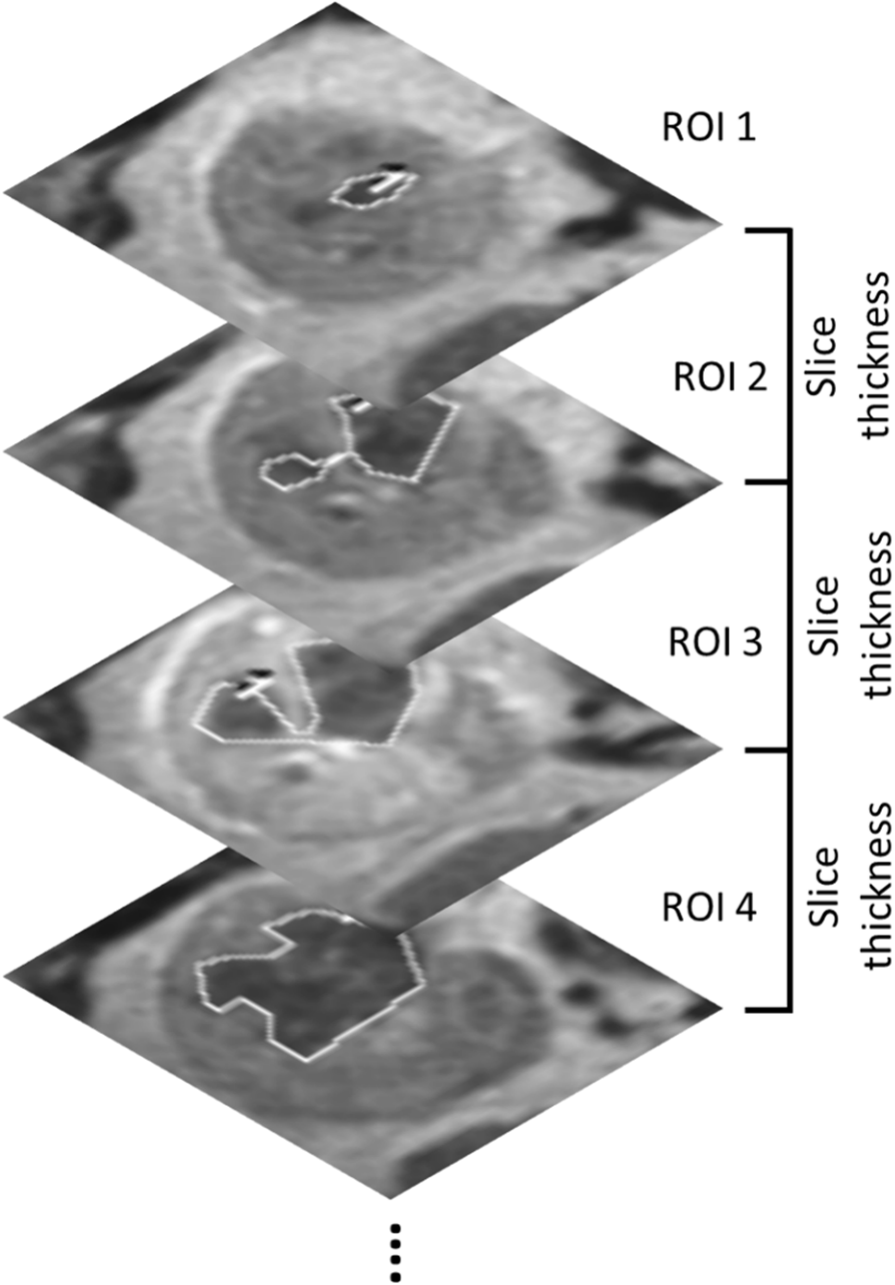
Diagram showing method for calculating volume of T2 low signal intensity area (T2LIA). Regions of interest (ROIs) are placed on T2-weighted images on every single slice of the visible tumor where there is measurable T2LIA. These ROIs are multiple by slice thickness and added together to calculated the volume of T2LIA. ROI = region of interest

The following MRI findings were assessed: (1) tumor size (> 10 cm vs. ≤10 cm) [17]; (2) ‘non-enhancing’ areas that may represent necrosis/hemorrhage [18, 19]; (3) lymphadenopathy defined as lymph nodes with a short-axis diameter > 1 cm or with suspicious features (e.g., avid enhancement, rounded appearance, heterogeneity, or irregular shape) [20]; and (4) volume of T2LIAs defined as discrete areas of low signal intensity on T2WI (compared with contralateral cortex), not corresponding to areas of hemorrhage [10, 14]. To measure the volume of T2LIAs, radiologists placed a region of interest on T2-WI on every slice that had visible T2LIA within the tumor on a PACS workstation (Centricity RA1000, GE Healthcare); these areas multiplied by the slice thickness were summed together to calculate the volume of T2LIA (Fig. 2). The entire tumor was also segmented to calculate the percentage of T2LIA defined as: volume of T2LIA / volume of tumor × 100.

### Clinicopathological data

The following information was obtained from the electronic medical records: age, gender, ethnicity, baseline metastatic status, details of pathological specimens (e.g., T stage, International Society of Urological Pathology grade, subtype, extent of sarcomatoid differentiation [focal vs non-focal]), treatments received, and follow-up (recurrence, metastasis, and death).

### Statistical analysis

Interobserver agreement between the radiologists was assessed with intra-class correlation coefficients (ICC) and Cohen’s Kappa (*k*), with the degree of agreement classified as: 0.00–0.39, poor, 0.40–0.59, fair, 0.60–0.74, good, and 0.75–1.00, excellent [21, 22]. The consensus MRI interpretation was used for the rest of the analyses. The Wilcoxon test was used to compare the volume and percentage of T2LIAs between patients that had focal vs. non-focal sarcomatoid differentiation on pathology. Kaplan-Meier analysis was used to calculate survival, defined as the time from MRI to death or last follow-up. Univariate and multivariate Cox proportional-hazards regression models were used to identify variables that were associated with survival. For multivariate analysis, some considerations were needed to avoid collinearity between the MRI and histopathological variables with similar information (e.g., tumor size, lymphadenopathy, and extent of sarcomatoid differentiation) [23]. We planned to use the one with greater significance for tumor size; MRI for lymphadenopathy since the extent of lymph node dissection was variable (none, standard vs extended) [24]; and MRI for extent of sarcomatoid differentiation since pathological analysis was done qualitatively. *P*-value < 0.05 was regarded statistically significant. R (version3.6.1, Vienna, Austria) was used for analysis.

## Results

### Patient and Clinicopathological characteristics

Table 1 describes the patient and clinicopathological characteristics. There were 59 patients (41 [69.5%] males and 18 [30.5%] females) with median age of 62 years (interquartile range [IQR] 51–68). Most patients were white (*n* = 47 [79.7%]). Twenty-six patients (44.1%) presented with metastatic disease at baseline. Patients underwent partial (*n* = 10 [16.9%]) or radical nephrectomies (*n* = 49 [83.1%]) after a median of 32 days (IQR 18–55) since MRI; lymph node dissection was performed in 42 patients (standard [*n* = 31] and extended [*n* = 11]) with median 8 nodes resected (IQR 6–18). Forty-five patients received neoadjuvant or adjuvant treatment, among which 37.8% (17/45) included an immune check point inhibitor: nivolumab (*n* = 7), ipilimumab+nivolumab (*n* = 8), or nivolumab+pembrolizumab (*n* = 2). All tumors were grade 4 by definition, and most were ≥ pT3 (*n* = 47, 79.7%). Median tumor size was 10.5 cm (IQR, 7.7–12.8). Extent of sarcomatoid differentiation was focal in 30 (50.8%) and non-focal in 29 (49.2%) patients. Histological subtypes were clear cell (*n* = 43 [72.9%]), papillary (n = 2 [3.4%]), chromophobe (*n* = 6 [10.2%]); remaining 8 (13.6%) patients had rare or unclassified histology. Metastatic lymph nodes were present in 13 (22.4%) patients.

**Table 1:** Clinicopathological and MRI characteristics of patients with sarcomatoid renal cell carcinoma

Characteristics	Category	No. of patients (%)^a^
**Clinicopathological findings**		
Age (years)		62 (51, 68)^a^
Gender	Male	41 (69.5)
	Female	18 (30.5)
Ethnicity	White	47 (79.7)
	Black	2 (3.4)
	Asian	6 (10.2)
	Unknown	4 (6.8)
Baseline metastatic status	Loco-regional	33 (55.9)
	Metastatic	26 (44.1)
Nephrectomy type	Radical	49 (83.1)
	Partial	10 (16.9)
Lymph node dissection	Standard	31 (52.5)
	Extended	11 (18.6)
	None	17 (28.8)
Systemic treatments^b^	None (only nephrectomy)	14 (23.7)
	Neoadjuvant only	2 (3.4)
	Neoadjuvant and adjuvant	6 (10.2)
	Adjuvant	37 (62.7)
Pathological tumor size (cm)		105 (7.7, 12.8)^a^
Pathological tumor stage	pT1	4 (6.8)
	pT2	8 (13.6)
	pT3	43 (72.9)
	pT4	4 (6.8)
Histological subtype	Clear cell	44 (74.6)
	Papillary	2 (3.4)
	Chromophobe	6 (10.2)
	Others^c^	7 (11.8)
Metastatic lymph nodes on pathology	Present	13 (22.4)
	Absent	46 (77.6)
**MRI findings**		
Size on MRI (cm)		10.5 (7.5, 12.7)^a^
Non-enhancing areas	Present	50 (84.7)
	Absent	9 (15.3)
Lymphadenopathy	Present	17 (28.8)
	Absent	42 (71.2)
T2LIA volume (ml)		3.0 (0.1, 13.0)^a^
Percentage of T2LIA (%)		0.8 (0.0, 2.9)^a^

^a^Presented as median and interquartile range; others are presented as number of patients and percentages

^b^Nivolumab alone (*n* = 7), ipilimumab and nivolumab (*n* = 8), or nivolumab and pembrolizumab (*n* = 2)

^c^Unclassified (*n* = 3), collecting duct type (*n* = 2) and fumarate-dehydratase deficient (*n* = 2)

### MRI findings

Agreement was excellent for tumor size (ICC = 0.99 [95%CI 0.97–0.99], volume of T2LIA [ICC = 0.84, 95%CI 0.74–0.91)]), and presence of non-enhancing areas (*k* = 0.86 [95%CI 0.60–1.00]) and good for lymphadenopathy (*k* = 0.73 [95%CI 0.48–0.98).

**Fig. 3:**
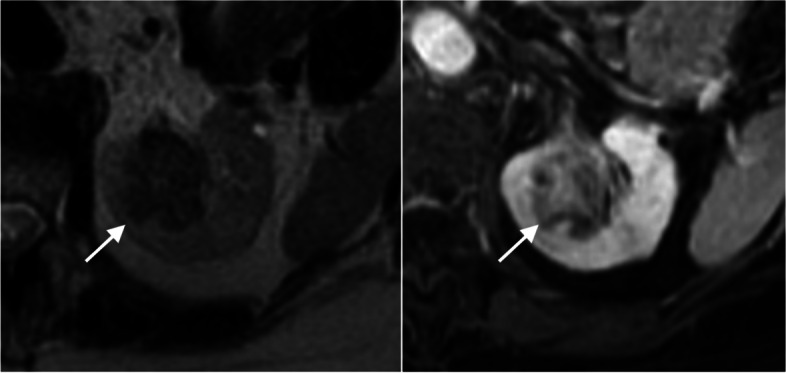
75-year-old woman with sarcomatoid RCC showing large volume of T2LIA. T2-weighted axial image (A) shows 3.7-cm left kidney endophytic mass (arrow) manifesting entirely with T2LIA (volume of 11.4 mL; 100%) that enhances on post-contrast T1-weighted image (B). Bone scan showed metastasis to left femur at baseline (not shown). Cytoreductive radical nephrectomy was done after which pathological analysis showed a 5.5-cm collecting duct type carcinoma with extensive sarcomatoid features and renal sinus fat invasion. Patient died 83 days after surgery before commencing further treatment. RCC = renal cell carcinoma; T2LIA = T2 low signal intensity area

**Fig. 4:**
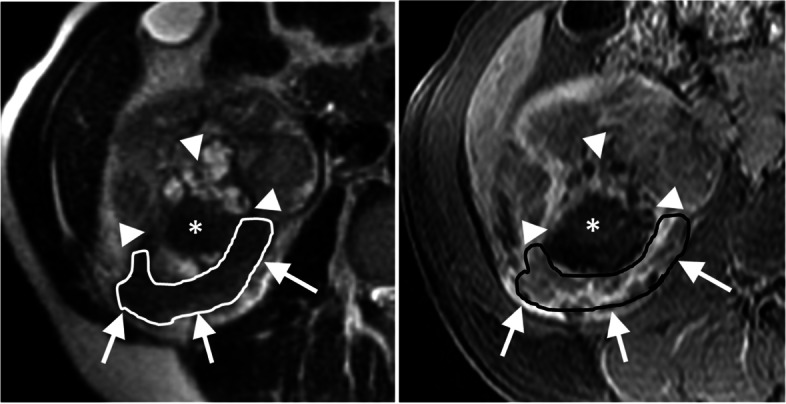
58-year-old man with sarcomatoid RCC showing small volume of T2LIA. T2-weighted axial image (A) and post-contrast T1-weighted image (B) shows 11.7-cm right kidney mass demonstrating small area T2LIA (arrows; volume of 15.2 mL; 2.5%) and adjacent non-enhancing area (arrowhead), probably representing hemorrhage (*) and/or necrosis. In the covered lung base, a 3.6-cm left lower lobe lung metastasis was noted (not shown). Cytoreductive radical nephrectomy was done after which pathological analysis showed a 11.5-cm clear cell RCC with extensive sarcomatoid differentiation and renal sinus fat invasion. Systemic treatment including sunitinib and gefitinib was started owing to progression of disease at the thoracic and abdominal lymph nodes, chest wall and lungs. Patient died 226 days after surgery. RCC = renal cell carcinoma; T2LIA = T2 low signal intensity area

**Fig. 5:**
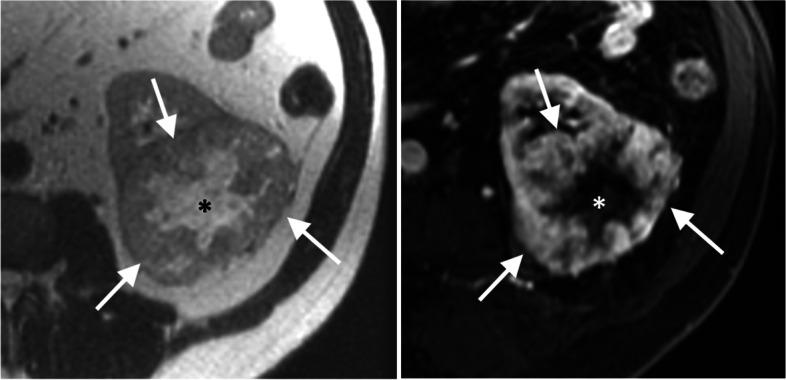
62-year old man with sarcomatoid RCC with no visible T2LIA. T2-weighted axial image (A) and post-contrast T1-weighted image (B) shows 8.5-cm avidly enhancing left kidney mass (arrows) with large central necrosis (*) but no measurable T2LIA. Baseline work-up revealed no metastatic disease. Radical nephrectomy was done after which pathological analysis showed 8.8-cm clear cell RCC with focal sarcomatoid differentiation and muscular venous branch invasion. After adjuvant treatment with pazopanib, patient developed left lower lobe lung metastasis 670 days after diagnosis and was subsequently treated with multiple lines of systemic treatment including lenvatinib, pembrolizumab, and nivolumab. Patient is still alive after 3397 days

Based on the consensus interpretation, median tumor size was 10.2 cm (IQR 7.2–12.8) among which 50.8% (*n* = 30) were larger than 10 cm. Nearly all patients had non-enhancing areas within the mass (*n* = 50 [84.7%]); lymphadenopathy was seen in 17 patients (28.8%). There was a measurable T2LIA in 72.9% (*n* = 43) of the patients with a corresponding median T2LIA volume and percentage of 3.0 mL (IQR 0.1–13.0) and 0.8% (IQR 0.0–2.9%), respectively. The median volume and percentage of T2LIA were significantly greater in patients that had non-focal than focal sarcomatoid differentiation on pathology (*p* < 0.01): volume, 8.0 mL (IQR 2.9–17.0) vs 0.7 mL (IQR 0.0–4.9); percentage, 2.8% [IQR 0.6–8.0] vs. 0.2% [IQR 0.0–1.4]). Representative cases are shown in Figs. 3, 4 and 5.

### Follow-up and survival

**Fig. 6:**
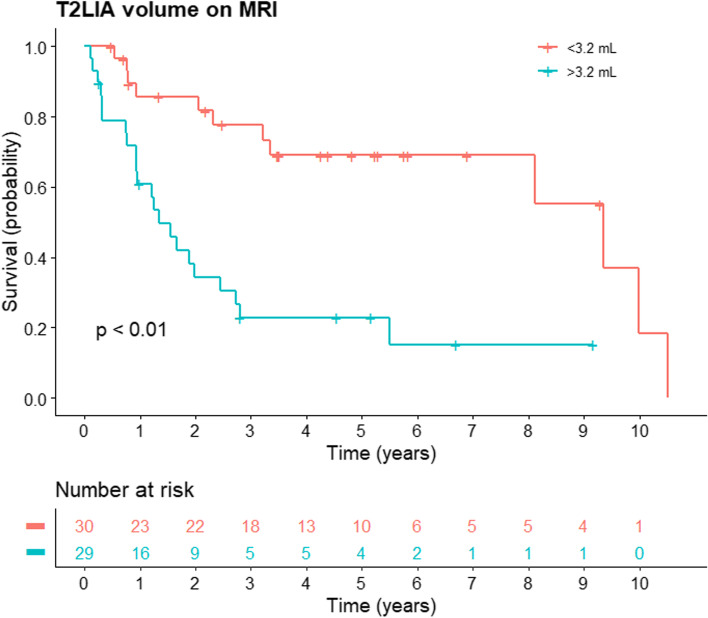
Kaplan-Meier survival curves stratified to volume of T2 low signal intensity area (T2LIA). MRI = magnetic resonance imaging

Thirty-four (57.6%) patients died after a median follow-up of 798 days (IQR 317–1709 days). Survival curves stratified by T2LIA volume is shown in Fig. 6 and those stratified by other MRI, clinical and pathological variables are respectively shown in Supplementary Figs. 1, 2, and 3; hazard ratios are summarized in Table 2. At univariate analysis, clinicopathological factors associated with shorter survival were: greater (> 10 cm) tumor size (HR = 2.44 [95%CI 1.15–5.21]; *p* = 0.02), metastatic lymph nodes (HR = 2.10 [95%CI 1.01–4.37]; *p* = 0.04), non-focal sarcomatoid differentiation (HR = 3.30 [95%CI 1.55–7.01]; *p* < 0.01), histologic subtypes other than clear cell, papillary, or chromophobe (HR = 3.25 [95%CI 1.28–8.20]; *p* = 0.01), and metastasis at baseline (HR = 5.04 [95%CI 2.40–10.59]; *p* < 0.01). Other variables including age, gender, ethnicity, pathologic tumor stage, and papillary or chromophobe subtypes were not associated with survival (*p* = 0.21–0.92).

**Table 2:** Hazard ratios of clinicopathological and MRI variables on uni- and multivariate survival analysis

Variable	Category	Univariate Analysis		Multivariate Analysis	
		HR (95% CI)	***P*** value	HR (95% CI)	***P*** value
**Clinicopathological findings**					
Age (years)	> 60	0.70 (0.34–1.43)	0.32		
	< 60	1			
Gender	Male	1.24 (0.55–2.79)	0.61		
	Female	1			
Ethnicity	Non-white	0.96 (0.41–2.23)	0.92		
	White	1			
Tumor Size (cm)	> 10	2.44 (1.15–5.21)	0.02	2.39 (0.90–6.37)	0.08
	< 10	1		1	
Histologic Subtype	Others	3.25 (1.28–8.2)	0.01	9.50 (2.81–32.13)	< 0.01
	Papillary or chromophobe	2.01 (0.68–5.95)	0.21	1.50 (0.47–4.74)	0.49
	Clear cell	1		1	
Extent of Sarcomatoid differentiation	Non-focal	3.30 (1.55–7.01)	< 0.01		
	Focal	1			
Pathologic Stage	pT3–4	2.20 (0.77–6.32)	0.13		
	pT1–2	1			
Metastatic Lymph Nodes	Present	2.10 (1.01–4.37)	0.04		
	Absent	1			
Baseline Status	Metastatic	5.04 (2.4–10.59)	< 0.01	6.89 (2.79–16.97)	< 0.01
	Loco-regional	1		1	
**MRI features**					
Tumor Size (cm)	> 10	1.96 (0.95–4.05)	0.07		
	< 10	1			
Volume of T2LIA (mL)	> 3.2	4.22 (1.92–9.29)	< 0.01	2.51 (1.04–6.05)	0.04
	< 3.2	1		1	
Percentage of T2LIA (%)	> 10%	2.11 (0.80–5.53)	0.12		
	< 10%	1			
Lymphadenopathy	Present	2.24 (1.16–4.71)	0.01	2.14 (1.00–4.57)	0.05
	Absent	1		1	
Non-Enhancing Areas	Present	0.64 (0.24–1.69)	0.37		
	Absent	1			

*CI* Confidence interval, *HR* Hazard ratio, *MRI* Magnetic resonance imaging, *T2LIA* T2 low signal intensity area

MRI features that were associated with shorter survival were: lymphadenopathy (HR = 2.24 (95%CI 1.16–4.71); *p* = 0.01) and volume of T2LIA (as a continuous variable, HR = 1.02 (95%CI 1.01–1.03; *p* < 0.01) and dichotomized by median (> 3.2 mL vs. < 3.2 mL, HR = 4.22 [95%CI 1.92–9.29]; *p* < 0.01). Presence of non-enhancing areas was not associated with survival (*p* = 0.37). Size and percentage of T2LIA showed a trend towards association with survival (*p* = 0.07 and 0.05, respectively).

At multivariate analysis, metastatic disease at baseline (HR = 6.89 [95%CI 2.79–16.97]; *p* < 0.01), other histological subtypes (HR = 9.50 [95%CI 2.81–32.13]; *p* < 0.01), and greater volume of T2LIA (HR = 2.51 [95%CI 1.04–6.05]; *p* = 0.04) remained independently associated with survival. Tumor size on pathology and lymphadenopathy on MRI showed a trend towards association with survival (*p* = 0.08 and 0.05, respectively).

In the 33 patients without metastatic disease at baseline, 18 (54.5%) developed recurrent or metastatic disease at a median follow-up of 364 days (IQR 154–450). The most common sites of metastases were lung (33.3% [11/33]), bone (27.3% [9/33]), and lymph nodes (21.2% [7/33]).

## Discussion

In this study, we evaluated the MRI findings of sarcomatoid RCC and their association with survival. We found that T2LIAs were seen in approximately three quarters of sarcomatoid RCCs and that the volume of T2LIA was independently associated with survival. Patients with T2LIA volume > 3.2 mL had shorter survival than those with T2LIA volume < 3.2 mL (adjusted HR = 2.51; *p* = 0.04). Our findings corroborate the observations of Takeuchi et al. [10, 14] which showed T2LIAs were characteristic of sarcomatoid RCC in that they correlate with the sarcomatoid component on pathology. Our results are also in agreement with a few studies that assessed the extent of sarcomatoid differentiation on pathology and found similar prognostic value [15–17]. Our study is the first to report the prognostic value of quantifying this imaging correlate of sarcomatoid differentiation on MRI, and therefore is unique in that it interconnects the fragmented literature discussing MRI findings and prognostic value of pathological analysis. Furthermore, given the poor prognosis of sarcomatoid RCC and the difficulties of managing patients with this rare tumor, we hypothesize that MRI may be a helpful adjunct tool providing additional information for improving prognostication in the management of these patients.

Early studies on T2LIA have been limited by the qualitative nature of assessing MRI features, which is susceptible to inter-reader variability, and underestimation of the extent of sarcomatoid involvement [14]. Interestingly, while T2LIA volume was significantly associated with shorter survival in our study, percentage of T2LIA was not. In fact, only 11.9% of the tumors demonstrated more than 10% of T2LIA, contrasting with the cohort studied by Adibi et al. [17] where percentage of sarcomatoid component on pathology greater than 10% was observed in 61.2%. This may be related to the presence of large non-enhancing areas which correlate with hemorrhage and necrosis which may render underestimation of T2LIA areas. Moreover, we specifically measured only the T2LIA that does not correspond to areas of hemorrhage, which often also manifests as T2 low signal on MRI, and hence leading to underestimation up to 19% as prior studies suggested [13]. Future studies should aim at identifying methods to better estimate the volume and percentage of sarcomatoid differentiation for it to better correlate with pathology and further advocate its value as a prognostic tool.

Tumor size and lymphadenopathy serve as additional predictors of survival in sarcomatoid RCC [5]. Sarcomatoid RCCs have been reported to be typically large, with a median tumor size of 9–10 cm [4, 25]. Our study is in agreement with this in that the median tumor size was 10.5 cm on pathology and 10.2 cm on MRI. Greater tumor size on pathology (HR = 2.44; *p* = 0.02) was associated with shorter survival on univariate analysis. Lymphadenopathy was seen on MRI in 17 patients (28.8%), yet 13 patients (22.4%) were recognized to have metastatic lymph nodes on pathology; both were associated with shorter survival at univariate analysis (HR = 2.24, *p* = 0.01 and HR = 2.10, *p* = 0.04, respectively) but with borderline significance at multivariate analysis (*p* = 0.05). In general, MRI has limitations for identifying lymph node metastases, even when using ancillary criteria (e.g., shape or enhancement) in addition to size criteria, both potentially missing micro-metastases and overdiagnosing reactive larger lymph nodes [26, 27]. Nevertheless, pathologic analysis of lymph nodes is also subject to variability from differences in the conduct of node dissection (none, standard, extended dissection) and whether or not the patient had undergone neoadjuvant therapies. These factors along with the small size of our cohort may have attributed to this borderline significance of lymphadenopathy when considering it has been shown to be predictive survival in a previous larger cohort of 207 patients [16].

Sarcomatoid RCC is known to not only present with more advanced stages of disease but is also associated with worse cancer-specific mortality than non-sarcomatoid RCC at each of the stages [28, 29]. Our results confirm this by demonstrating that almost half of the patients presented with baseline metastatic disease, which was associated with significantly poorer prognosis (adjusted HR = 6.89; *p* < 0.01). Even for patients with localized disease, recurrence or new metastasis often occurs within 2 years after surgery [5]. We too found that recurrent or metastatic disease occurred in more than half of the patients who did not have metastasis at baseline after a median 1 year follow-up. In addition, we affirmed that the majority of sarcomatoid dedifferentiation occurred from clear cell RCCs, consistent with prior observations [3]. We also found that subtypes other than clear cell, papillary, and chromophobe were associated with shorter survival. This is speculated to be attributed to the fact that these were unclassified, collecting duct type, or associated with fumarate dehydratase deficiency [30]. Moreover, sarcomatoid RCC tends to present more often in males and at ages 54─63 years; however, their associations with survival and have not been well-studied [5, 6, 31]. In our study, age, ethnicity, and gender were not associated with survival.

There were a few limitations in our study. First, it was based on a small number of patients at a single institution, done retrospectively. Although most patients with renal masses will undergo cross-sectional imaging, MRI is more commonly done in certain circumstances (e.g., problem-solving when CT is equivocal). Second, interpretation of outcomes should also take into consideration the heterogeneity of systemic therapies. The evolving therapeutic landscape over the past two decades can be reflected in the substantial increase in immune checkpoint inhibitors used: 4.2% during earlier two thirds vs 50% during the later one third of the inclusion period. Moreover, sarcomatoid RCC has generally been resistant to conventional treatments, although combination therapies with chemotherapy and targeted or immunotherapy have been able to improve survival in some patients [5, 32]. Third, we were not able to perform a radiologic-pathologic correlation of the sarcomatoid component. Moreover, the extent of sarcomatoid differentiation on pathology was qualitatively assessed.

## Conclusion

Sarcomatoid RCCs were generally large and demonstrated non-enhancing areas suggestive of necrosis and hemorrhage on MRI. T2LIA, an imaging correlate for sarcomatoid differentiation, was seen in most of the patients and its volume was independently associated with survival along with histological subtype and baseline metastatic status.

## Supplementary Information

**Additional file 1: Supplementary Fig. 1.** Kaplan-Meier survival curves stratified to other MRI findings. T2LIA = T2 low signal intensity area. MRI = magnetic resonance imaging.

**Additional file 2: Supplementary Fig. 2.** Kaplan-Meier survival curves stratified to clinical variables.

**Additional file 3: Supplementary Fig. 3**. Kaplan-Meier survival curves stratified to pathological variables.

## Data Availability

The datasets used and analyzed for the current study are available from the corresponding author upon reasonable request.

## Abbreviations

MRI: Magnetic resonance imaging
RCC: Renal cell carcinoma
T2LIA: T2 low signal intensity area
T2WI: T2-weighted imaging
ISUP: International Society of Urological Pathology
IQR: Interquartile range
