# Supplementary figures and images for: Sarcomatoid renal cell carcinoma: MRI features and their association with survival
Source: Cancer Imaging. 2023 Feb 15;23:16. doi: 10.1186/s40644-023-00535-0 (PMC9930281; doi:10.1186/s40644-023-00535-0)

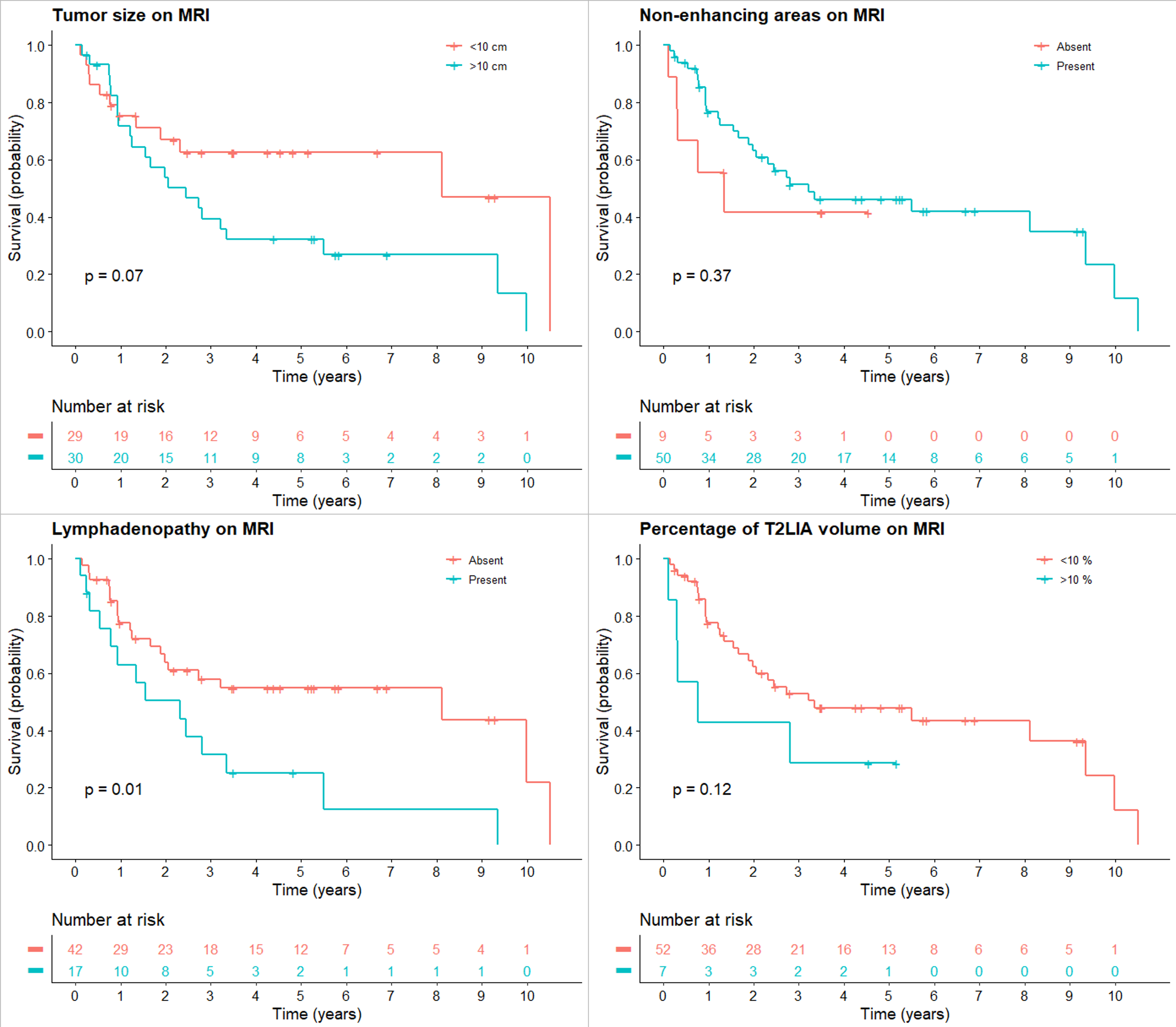

Supplement: Supplementary file 1 — Additional file 1: Supplementary Fig. 1. Kaplan-Meier survival curves stratified to other MRI findings. T2LIA = T2 low signal intensity area. MRI = magnetic resonance imaging. [file 40644_2023_535_MOESM1_ESM.tif]

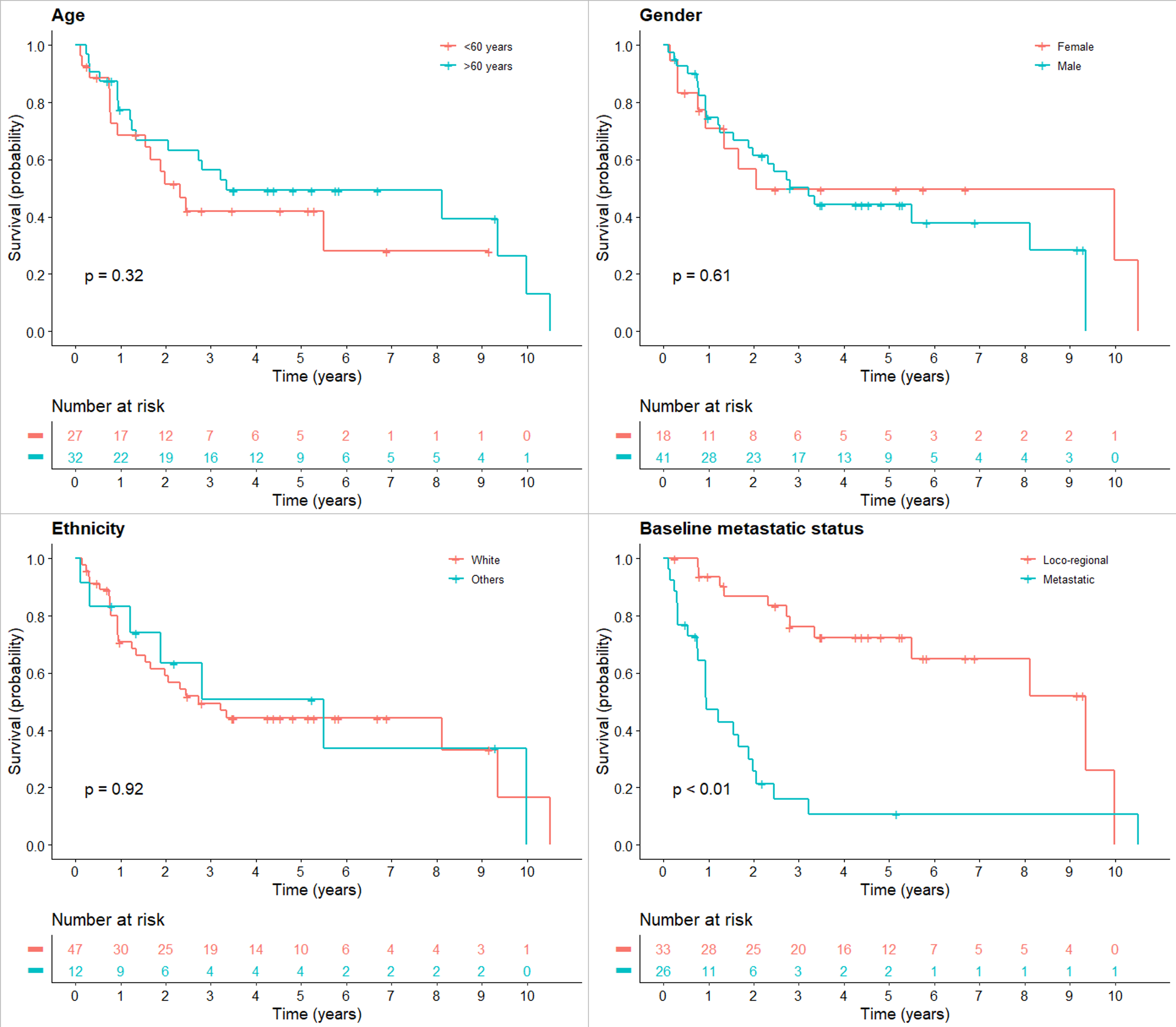

Supplement: Supplementary file 2 — Additional file 2: Supplementary Fig. 2. Kaplan-Meier survival curves stratified to clinical variables. [file 40644_2023_535_MOESM2_ESM.tif]

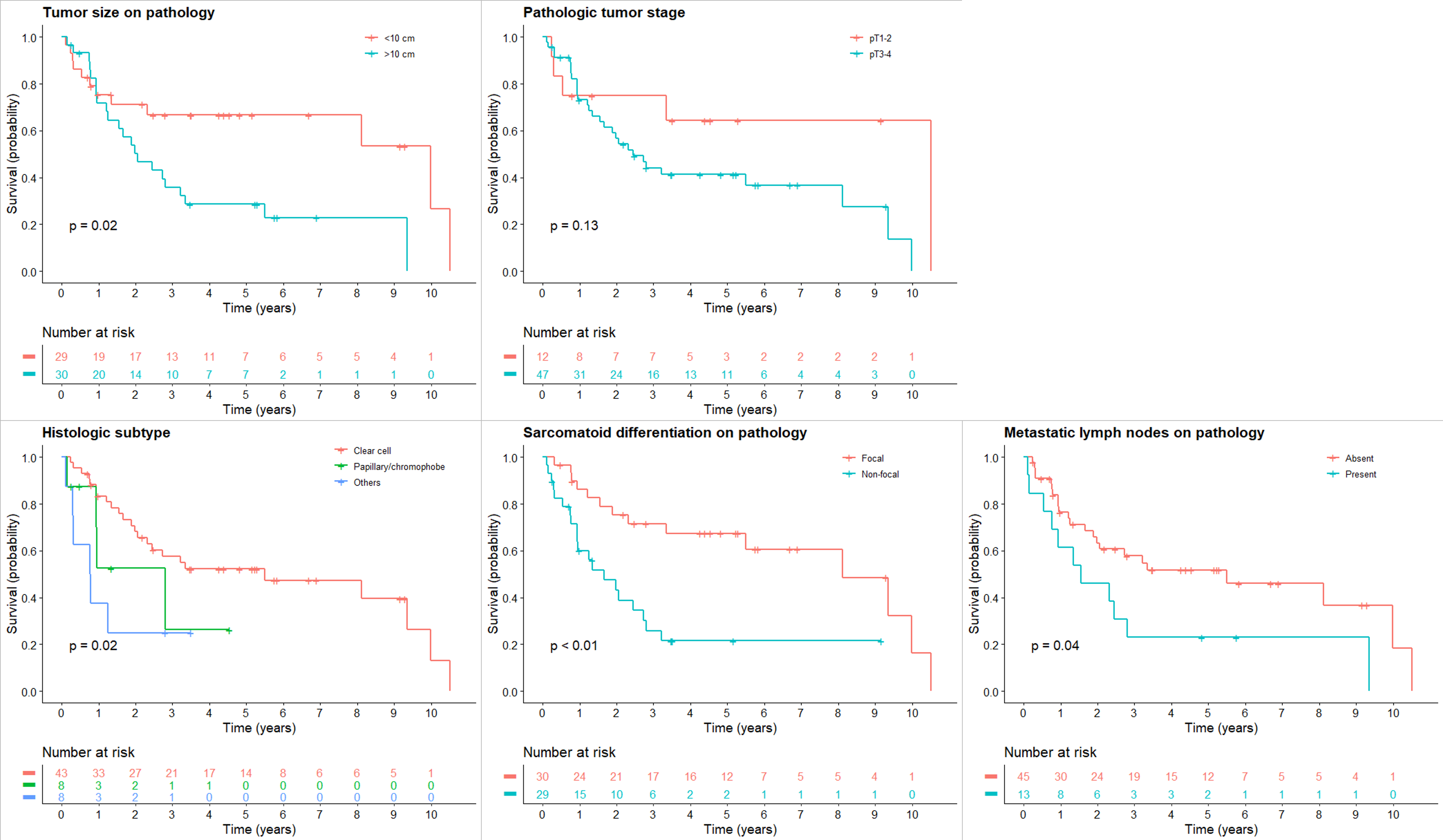

Supplement: Supplementary file 3 — Additional file 3: Supplementary Fig. 3. Kaplan-Meier survival curves stratified to pathological variables. [file 40644_2023_535_MOESM3_ESM.tif]
